# The History of the Molybdenum Cofactor—A Personal View

**DOI:** 10.3390/molecules27154934

**Published:** 2022-08-03

**Authors:** Ralf R. Mendel

**Affiliations:** Institute of Plant Biology, Technical University Braunschweig, Humboldtstrasse 1, 38106 Braunschweig, Germany; r.mendel@tu-bs.de; Tel.: +49-(0)160-9085-6244

**Keywords:** molybdenum, molybdenum cofactor biosynthesis, molybdopterin, nitrate reductase, gephyrin

## Abstract

The transition element molybdenum (Mo) is an essential micronutrient for plants, animals, and microorganisms, where it forms part of the active center of Mo enzymes. To gain biological activity in the cell, Mo has to be complexed by a pterin scaffold to form the molybdenum cofactor (Moco). Mo enzymes and Moco are found in all kingdoms of life, where they perform vital transformations in the metabolism of nitrogen, sulfur, and carbon compounds. In this review, I recall the history of Moco in a personal view, starting with the genetics of Moco in the 1960s and 1970s, followed by Moco biochemistry and the description of its chemical structure in the 1980s. When I review the elucidation of Moco biosynthesis in the 1990s and the early 2000s, I do it mainly for eukaryotes, as I worked with plants, human cells, and filamentous fungi. Finally, I briefly touch upon human Moco deficiency and whether there is life without Moco.

## 1. Introduction

### 1.1. Molybdenum

It has long been known that the transition element molybdenum (Mo), with its rich redox chemistry, is an essential micronutrient for plants, animals, and microorganisms where it forms part of the active center of enzymes. It is the only fifth-row element with biological function and occurs in soil and the oceans as an oxide or sulfide compound [1]. Mo is a trace element, i.e., the organism needs it only in minute amounts, but the unavailability of Mo is detrimental to the organism. However, even if Mo is available for the cell, it is biologically inactive until it becomes complexed to form the molybdenum cofactor. Nature has developed two very different cofactors to position Mo within the active center of Mo enzymes. One type of molybdenum cofactor is the iron–molybdenum cofactor (FeMoco) which is unique to a single enzyme, bacterial nitrogenase (FeMoco is reviewed in detail by Ribbe et al. in this Special Issue). The other type is the pterin-based molybdenum cofactor (Moco), which occurs in all kingdoms of life. Both cofactors have in common that the metal atom is coordinated by sulfur—likely due to the sulfur-rich environment in the ancient ocean. Calculations of molecular evolution show that both cofactors were already present in the last universal common ancestor LUCA [2,3], indicating their essential role for life. Mo is abundant in the oceans in the form of the molybdate anion (about 100 nM [4]). In soils, the oxidation state of Mo varies from II to VI, but only the soluble molybdate anion is available for eukaryotes and bacteria.

### 1.2. Molybdenum Enzymes

Mo enzymes and Moco are found in all kingdoms of life, where they perform key transformations in the metabolism of nitrogen, sulfur, and carbon compounds [1]. They have a very rich redox chemistry and each reaction, either reduction or oxidation, is also characterized by the transfer of two electrons, forcing the Mo atom to vary its oxidation state between IV and VI. More than 50 Mo-containing enzymes are known, most of them of bacterial origin, although only a handful of Mo enzymes were found among eukaryotes. In brief, these eukaryotic enzymes are (1) sulfite oxidase, which catalyzes the final step in the degradation of sulfur-containing amino acids and is involved in detoxifying excess sulfite; (2) xanthine dehydrogenase, which is involved in purine catabolism and reactive oxygen production; (3) aldehyde oxidase, which oxidizes a variety of aldehydes and is essential for the biosynthesis of the stress hormone abscisic acid in plants; (4) nitrate reductase in autotrophic organisms, which catalyzes the crucial step in inorganic nitrogen assimilation; and (5) mitochondrial amidoxime reductase which has a detoxifying function. The structures and functions of all five enzymes are reviewed by Hille et al. (2011) [5]. In bacteria, well-studied Mo enzymes are formate dehydrogenase, nitrate reductase, DMSO-reductase, xanthine dehydrogenase, and aldehyde oxidase. In extremophile bacteria and archeae, Mo is replaced by the transition element tungsten [6]. 

In this review, I focus on Mo; details for tungsten can be found in the comprehensive review by Bevers et al. [7]. I started as a plant researcher; therefore, in my review, I focus on the history of eukaryotic Moco research, mainly in plants and humans, because these organisms are drivers in the field. Details for the history of bacterial Moco research are given by Leimkühler et al. [8].

## 2. History of Moco

### 2.1. 1900–1954: Mo Research Started with Cow Milk

As early as 1889, it was observed that xanthine could be converted to uric acid in tissue extract [9]. In 1902, Schardinger [10] found that a protein fraction of fresh cow milk could reduce methylene blue in the presence of formaldehyde. This protein fraction was also able to oxidize other aldehydes and became known as Schardinger’s enzyme. In 1922, Morgan et al. [11] identified the reducing substance as hypoxanthine and named the enzyme xanthine oxidase. In 1949, Westerfeld and Richert [12] found that for xanthine oxidase activity in addition to FAD, an unknown compound was necessary that was primarily found in liver extracts, and named it “liver residue factor” [12]. Two years later, these researchers renamed this factor “xanthine oxidase factor” and identified it as Mo [13]. In the same year, DeRenzo et al. [14] also identified Mo as the xanthine oxidase factor. Shortly after, Mo was also found in aldehyde oxidase [15] and nitrate reductase [16], and later in sulfite oxidase [17]. However, the occurrence of Mo in living organisms had already been described earlier—in 1930, Bortels [18] described Mo as catalytically crucial for the biological nitrogen fixation in the bacterium *Azotobacter*, and in 1932, Ter Meulen [19] chemically identified Mo in plants (especially rich in legumes) and mammals (especially rich in the liver).

### 2.2. The 1960s: Moco Was Postulated by Geneticists

In algae, fungi, and higher plants, the Mo enzyme nitrate reductase (NR) catalyzes the key step in inorganic nitrogen assimilation, and NR-deficient mutants were soon described. With the genetic analysis of these mutants, the genetic elucidation of Mo metabolism had begun, namely, in the filamentous fungus *Aspergillus nidulans* by Pateman et al. [20]. Cove and Pateman [21] had isolated NR-deficient mutants which revealed a novel phenotype: the simultaneous loss of the two Mo-dependent enzymes NR and xanthine dehydrogenase. Mo was the only link between these two otherwise very different enzymes; thus, it was suggested that both enzymes could share a common Mo-related cofactor, named molybdenum cofactor. Pateman et al. [20] described five genetic loci, each exhibiting that novel phenotype, and designated them with the mnemonic ‘Cnx’ (cofactor for nitrate reductase and xanthine dehydrogenase). Thus, it became evident that for this common cofactor, five genes were responsible, whereas for nitrate reductase, only one locus was coding. 

One decade later, Cnx-like mutants were described for the plant *Nicotiana tabacum* [22]; two decades later, Cnx-like mutants were published for the fly *Drosophila melanogaster* [23]; and three decades later, Cnx-like mutants were defined for other higher plants, among them, the model plant *Arabidopsis thaliana* [24].

In parallel to the achievements of fungal biochemical genetics, also in *Escherichia coli*, a series of mutants had been isolated that showed the growth of mutagenized cells in the presence of high chlorate concentrations. Mutants selected for chlorate resistance (*chl*) do not reduce chlorate to toxic chlorite because they have lost their chlorate reductase activity, which appears to be a non-physiological catalytic activity related to NR [25,26]. We now know that the chlorate-resistant phenotype reflects the lack of NR activity, either due to a mutation in the corresponding structural genes or to a loss of Moco. However, at that time, it was surprising that many independent genes were involved in the loss of NR. This observation had already been made by Aspergillus researchers a few years earlier. Later, it turned out that the chlorate-resistant loci *chlA*, *chlB*, *chlD*, *chlE*, and *chlG* were all essential for Moco biosynthesis. Stewart and MacGregor [27] isolated an extensive series of Mu-phage insertion mutants for all these loci. In 1992, the Moco-specific *chl* loci were renamed *mo* loci [28].

### 2.3. The 1970s: The ‘Nit–1 Assay’ and the Discovery of Moco

In the filamentous fungus *Neurospora crassa*, Sorger and Giles [29] isolated a mutant named *nit-1*, similar to the Aspergillus Cnx mutants, exhibiting a pleiotropic loss of NR and xanthine dehydrogenase activities. In 1970, Nason and coworkers [30,31,32] provided the first biochemical evidence for the existence of a cofactor common to Mo enzymes. In crude extracts of *nit-1* mutant cells, the inactive apoprotein of NR could be fully reconstituted by adding a low-molecular-weight fraction derived from denatured preparations of purified Mo enzymes of mammalian, plant, or bacterial origin. This fraction included a Mo-containing cofactor that could be integrated into inactive apoNR of the fungal *nit-1* mutant lacking the cofactor. Thus, it was suggested that a ubiquitous and universal Moco of identical structure could associate with different apoenzymes to form Mo holoenzymes. However, there was one exception: nitrogenase, another Mo-containing enzyme, did not release a *nit-1* positive cofactor after denaturing the enzyme [33]. Based on these data and functional analyses [34], it was shown that the dissociable cofactor of nitrogenase, named the FeMo cofactor, is different from Moco and unique for nitrogenase (see the review by Ribbe in this Special Issue).

The observed complementation of *nit-1* apoNR by Moco from different Mo enzyme sources served as a basis to develop a very sensitive biological in vitro assay, referred to as the ‘*nit-1* assay’, for determining the presence of biologically active Moco, which still serves as a tool for Moco analysis. Nason et al. [35] found that the reconstituting activity released from diverse Mo–enzymes was very labile, with a lifetime of a few minutes. This was the first indication of the cofactor’s labile nature in its isolated form. Subsequently, several attempts were undertaken to stabilize the cofactor and to optimize the transfer of Moco to *nit-1* apoNR.

#### 2.3.1. Release of Moco from Mo Enzymes under Stabilizing Conditions

Different methods of cofactor release from Mo enzymes (acidification [32]; heat treatment [36]; detergent and organic solvent treatment [37,38]; phosphate extraction [39]) were utilized to minimize the loss of cofactor due to its high sensitivity to air oxidation. Most of these in vitro complementations are dependent on an excess of molybdate (1–10 mM) in the reaction mixture [40,41,42,43], indicating a strong tendency of isolated Moco to lose Mo. Furthermore, it had been shown through the *nit-1* assay that different sulfhydryl protecting agents stabilize the isolated cofactor [36,41], with glutathione exhibiting the best results, whereas sulfhydryl-reactive inhibitors such as *p*–hydroxymercuribenzoate abolished the reconstituting activity of the cofactor [42,44], thus indicating the involvement of free sulfhydryl groups in the *nit-1* reconstitution process.

#### 2.3.2. Moco Triggers the Dimerization of NR Monomers

Nason and coworkers [31] also observed another attractive property of Moco. In extracts of the *Neurospora nit-1* mutant, inactive apo-NR occurs in monomeric form, sedimenting with 4.5S in sucrose density gradients. After adding Moco released from Mo enzymes to the *nit-1* extract for in vitro complementation, NR gained activity and sedimented as a dimer with 7.9S. Moco triggered a clear conformational change in the NR monomer so that it could dimerize.

Although an absolute quantification of Moco by the *nit-1* assay was hard to achieve, it has been beneficial for qualitative and semi-quantitative detection of cofactor activity. Later, this in vitro system was improved: *Neurospora crassa* Moco-free apo-NR had been cloned, tagged (twin-Strep and His), and the purified apo-NR could be used to quantify Moco [45]. This study also showed that Moco insertion into apo-NR is independent of the insertion of the two other prosthetic groups, FAD and heme. The formation of active holo-NR is an autonomous process intrinsically tied to NR dimerization. There is now a chemical assay to quantify Moco [46]. However, this assay based on the chemical detection of the pterin moiety of Moco does not allow for the detection of biologically active Moco. Therefore, the *nit-1* assay is still the most straightforward way to detect biologically active Moco in crude extracts of diverse biological sources.

### 2.4. The 1980s: The Elucidation of the Chemical Nature of Moco

#### 2.4.1. Moco Degradation Products Were Crucial for Uncovering Its Chemical Nature

**Figure 1:**
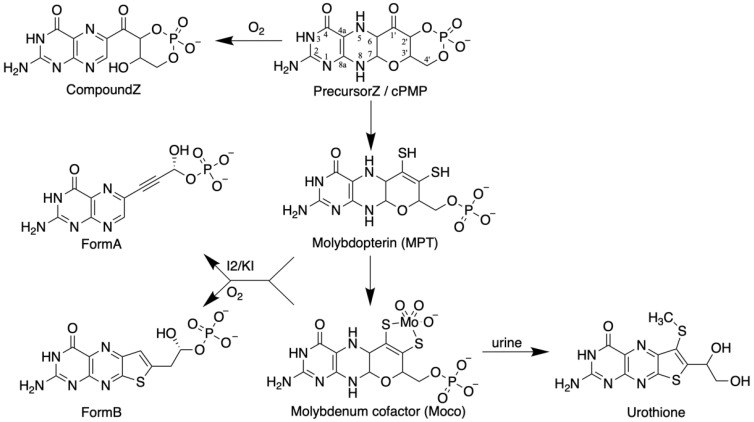
Structures of PrecursorZ, molybdopterin (MPT), Moco, and their derivatives. PrecursorZ is cyclic pyranopterin monophosphate (cPMP), and CompoundZ is its air-oxidized product. MPT and Moco are both 5,6,7,8-tetrahydropyranopterins. FormB is the air-oxidized degradation product of MPT and Moco, whereas FormA is the iodine-oxidized derivative. FormA and FormB are shown in their phosphorylated forms. Urothione is the degradation product of Moco in humans, which is excreted in the urine.

The elucidation of the chemical nature of Moco is based on the work of J. Johnson and K.V. Rajagopalan (Figure 1). Spectroscopic analyses of Moco isolated from chicken and rat livers yielded a pterin component [37]. Due to the labile nature of Moco and its high sensitivity to air oxidation, all further studies were conducted using degradation or oxidation products of the cofactor isolated from the liver. By oxidizing Moco under acidic conditions, two fluorescent pterin derivatives were found (FormA and FormB, Figure 1), which were crucial to solving the structure of Moco. In short, surprisingly, FormA contained a four-carbon atom side chain at its C6, which is uncommon for pterins because most of them have three carbons in their side chain [47]. However, no suitable ligand groups were found as candidates for binding Mo in FormA. Due to the sensitivity of Moco to sulfhydryl reagents and its increased stability in the presence of reducing reagents, the presence of sulfur atoms in the cofactor was assumed. Here, FormB led the way because it contained a sulfur atom. At that point, it must be mentioned that as early as 1940, Koschara [48] described a non-fluorescent sulfur-containing pterin named urothione in human urine. Goto et al. [49] determined the structure of urothione, which turned out to be a sulfur-containing pterin compound (Figure 1). The Rajagopalan group finally showed that the intense oxidative treatment of FormB and urothione yielded the same compound; thus, the conclusion was very suggestive that urothione may be the natural metabolic degradation product of Moco. Conclusive evidence for this came from the urine analysis from patients with Moco deficiency devoid of urothione [50]. The coordination of Mo via an enedithiolate function at the C1’ and the C2’ position in the four-carbon side chain of the cofactor was demonstrated so that the structure of Moco was suggested to be as shown in Figure 1 [50]. All details of this chemical detective story were described by Leimkühler et al. [8]. Finally, crystal structures of Mo enzymes confirmed this core structure [51] and showed a third pyrano ring between the OH-group at C3’ of the side chain and the pterin C7 atom in its 5,6 dihydro state. Once the pyrano ring is closed, a fully reduced hydrogenated pterin is formed. Due to the unique nature of the pterin in Moco, the metal-free form of the cofactor is called molybdopterin, or metal-binding pterin (MPT). The latter reflects that this pterin scaffold cannot only coordinate Mo, but also tungsten.

Later, it was found that in bacteria, a dinucleotide derivative of Moco exists where the MPT moiety of Moco is associated with the nucleotide GMP or CMP, and in most Mo enzymes occurred in the bis-form where two MPT dinucleotides coordinated one Mo atom (details are given in the review by Tiedemann et al. [52] in this Special Issue).

#### 2.4.2. What Is the Task of the Pterin Moiety of Moco?

The fusion of a pterin with a pyrano ring, as identified for Moco and its direct precursor, the metal-free MPT, is unique in nature. It may have been evolved to serve the following possible tasks: (a) positioning the catalytic metal correctly within the active center of a given Mo enzyme, and depending on the class of Mo enzyme, Mo can have different ligands [1]; (b) another possible role of the pterin moiety could be modulation of the redox behavior of the Mo atom; (c) pterin might also participate in the electron transfer to or from Mo via delocalized electrons within the pterin; (d) with its possible reduction states and different structural conformations, the pterin could also be necessary to channel electrons to or from other prosthetic groups [1]. Moco is not located on the surface of the protein. Rather, X-ray crystallographic analyses of Mo enzymes revealed that the cofactor is buried deeply within the interior of the enzyme and a tunnel-like structure makes it accessible to both its appropriate substrates and interacting prosthetic groups [53,54,55].

#### 2.4.3. Chemical Model Compounds Helped to Study Moco

Two lines of research converged after the chemical nature of Moco was determined: dithiolene chemistry and pterin chemistry. The research field of metal 1,2-dithiolene chemistry started in the 1960s [56]. After it became clear that Moco is a unique Mo–dithiolene–pterin compound, the synthesis and study of Mo–dithiolene complexes began with the aim of reproducing the general features of the Mo coordination sphere. The detailed chemical and spectroscopic analyses of these synthetic analogs of catalytic centers in Mo enzymes gave insight into Moco’s broad redox capabilities [57]. The Mo–dithiolene group, however, is only one part of Moco; the other component is its pterin scaffold. Pterin chemistry is a historic research field deriving from the analysis of natural products. Pterins are ubiquitous in nature. They have been identified as pigments in the wings of butterflies, which was name-giving: the Greek word for wing is ‘pteron’. They are C1 transfer cofactors (folic acid and tetrahydrofolates), redox cofactors (biopterin and neopterin), and even toxins in mollusks. Pterins are of great medical relevance; therefore, they are extensively studied in chemistry and biochemistry, life sciences, medicine, pharmacology, and the pharmaceutical industry. Chemical synthesis of pterins began in the 1940s (see the review by Basu and Burgmayer [58]). Additionally, soon after the identification of Moco as a pterin derivative, pterin-inspired model chemistry started with the synthesis of pterin-substituted dithiolenes [58]. There are ongoing attempts for the total chemical synthesis of Moco [59,60]. However, it has not yet been achieved, although the chemical synthesis of Moco’s precursor, cPMP, has been successful [61]. For a recent review, see Colston and Basu [62] in this Special Issue. In parallel to these achievements, another very active field of research has developed to reproduce the reactions catalyzed by Mo enzymes in vitro. Bio-inspired chemistry has emerged in this field and is also used as a basis for the design of biocatalysts to address contemporary issues.

### 2.5. The 1970s and 1980s: Biochemical Analyses of Moco Mutants in Bacteria, Fungi, and Plants Gave the First Insights into Moco Biosynthesis

The detailed biochemical characterization of NR-deficient mutants contributed significantly to our understanding of the biochemistry and genetics of Moco biosynthesis in bacteria, fungi, and plants. All of them exhibited no NR activity; thus, the question arose as to how to find other criteria for differentiating between them?

#### 2.5.1. Repair by High Molybdate

It is reasonable to assume that the multitude of mutant loci in each model organism are involved in Moco metabolism; a first and easy experimental step was to grow the different NR-deficient mutants on a high-molybdate medium (1 mM): among the *Aspergillus nidulans* Cnx mutants, one locus turned out to be partially molybdate-repairable [63]; in the plant *Nicotiana tabacum*; one mutant type was also partially molybdate-repairable [40]. Among newly described *Neurospora crassa nit* loci [64], again, mutants in one locus were molybdate-repairable [40]. Additionally, among the *E. coli chl* mutants, two groups were molybdate-repairable, although the situation is more complex (see below). Thus, it was assumed that the gene products of the molybdate-repairable locus should be involved in transferring or inserting Mo into MPT. Subsequent molecular analyses proved this assumption to be correct.

#### 2.5.2. What Was Missing in *E. coli* Chlorate Mutants?

*E. coli* NR was intensively studied in the 1960s and 1970s, and after the chlorate-resistant mutants had been published [25,65], which all lacked NR activity, it became of interest to find out what was missing in these mutants. How could criteria other than high molybdate repair be found for differentiating between them?

At first, cell-free extracts of *chl* mutants were mixed and analyzed for the reconstitution of NR activity. Later, protein fractions were purified, containing the reconstituting activities and resulting in the initial characterization of ‘Protein FA’ [66] and ‘Protein PA’ [67], which turned out to be encoded by *chl* loci. Using the *Neurospora nit-1* assay (described above), the extracts of the mutants *chlA*, *chlB*, *chlC*, *chlD*, *chlE*, and *chlG* were analyzed in combination with additional molybdate in the assay, providing an initial suggestion about the sequence of activities of the *chl* gene products. Two loci were repairable by high molybdate (*chlD* and *chlG*), and two were assumed to act early in Moco biosynthesis (*chlA* and *chlE*) because they showed no *nit-1* complementing activity. *chlB* was assumed to be involved in the insertion of Moco in apoNR [68,69]. Using the *nit-1* assay, Miller and Miller and Amy [69] found that in *chlG* mutants, a demolybdo form of the cofactor (MPT) was present, which incorporated Mo into MPT only in the presence of high molybdate (10 mM). An additional factor named factor X was identified by in vitro complementation, essential for the formation of the active NR complex in *E. coli*. Two decades later, this enigmatic factor turned out to be the chaperone narJ, the first of a long list of specific chaperones assisting in the folding and acquisition of metal cofactors, including Moco.

#### 2.5.3. Plant Mutants Disclose Further Details about Moco Biosynthesis

In the 1970s, in our laboratory, cell cultures of the model plant *Nicotiana tabacum* were mutagenized and selected for chlorate resistance [70]; I was assigned the task of biochemically analyzing them during my Ph.D. research [22,71,72]. All of them had lost NR activity, and some of the selected cell lines exhibited no xanthine dehydrogenase activity [22], thus representing putative Moco mutants. We chose to name them with the initialism ‘Cnx’ [72], which stands for cofactor for nitrate reductase and xanthine dehydrogenase, similar to the *Aspergillus* geneticists’ suggestion [20]. I used a number of different criteria to further biochemically classify these Cnx mutants:

- By measuring intracellular amounts of molybdate, it could be ruled out that plant Moco mutants are defective in molybdate uptake [41,73];
- Running the extracts of plant Cnx mutants on sucrose density gradients and measuring the diaphorase activity of NR (i.e., their cytochrome *c* reductase activity which is independent of Moco) identified one mutant type that had dimeric NR [72];
- Additionally, when this mutant was subjected to the *nit-1* assay, it was positive, i.e., it could synthesize MPT [40];
- As this mutant type regained NR activity when grown on high molybdate [40] or when molybdate was added to crude extracts of the cells [74,75], it was assumed to be impaired in molybdate insertion into MPT, which should be a late step in Moco biosynthesis;
- Other mutants were negative in the *nit-1* assay (i.e., were unable to synthesize MPT), but they could be repaired in vitro by adding Moco released from bovine xanthine oxidase [74]. Those mutants were classified to be defective in the early steps of Moco synthesis;
- As another differentiating criterion, I used Moco fluorescence—cell extracts of the Cnx mutants were heat-treated [36,76] to release Moco, and a fluorescence spectrum was recorded. Again, only the molybdate-repairable mutant type described above was positive [74].

In summary, six independent Cnx mutants (CnxA–CnxF) were identified in the model plant *Nicotiana plumbaginifolia* [77,78]. Additionally, in other plants (e.g., barley, pea, *Arabidopsis thaliana* (reviewed by Crawford [24]) and in the alga *Chlamydomonas reinhardtii* (cf. review [79]), Moco mutants were isolated.

### 2.6. 1992: The First Model for the Moco Biosynthesis Pathway in E. coli

**Figure 2:**
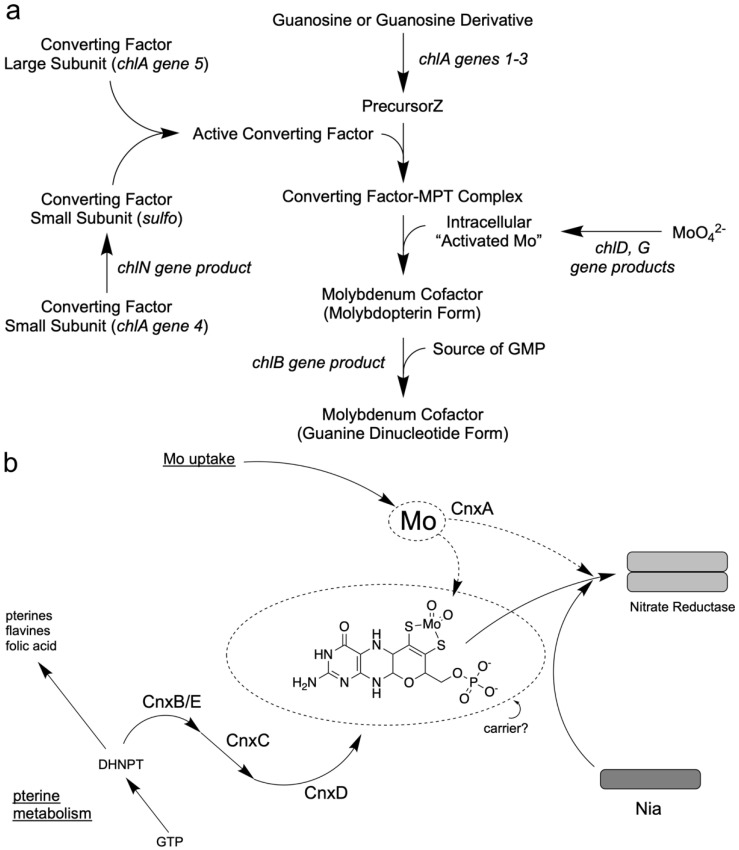
Models for the Moco biosynthesis pathway. (**a**) Model for the Moco biosynthesis pathway in *E. coli*, published by the Rajagopalan group in 1992. The proteins are named according to their functions. Modified after [81]. (**b**) Model for the Moco biosynthesis pathway in plants, published by Mendel in 1992 [84]. The proteins are named in the Cnx nomenclature. CnxA is the molybdate insertase. DHNPT stands for dihydro neopterin triphosphate. Nia is the designation for the nitrate reductase monomer. Modified after [84].

Comprehensive analyses of the *E. coli chl* mutants involving genetic and biochemical studies by several laboratories led to a working model for Moco biosynthesis. In the above section, “What was missing in *E. coli* chlorate mutants?” I referred to the proteins “Association factor FA” [66] and “Protein PA” [67] which were isolated and initially characterized from *E. coli* cell extracts by French groups and that were products of the *chl* genes. In 1990, Hinton and Dean [80] presented an initial model where they included all previous biochemical and genetical efforts undertaken to reveal insights into Moco biosynthesis in *E. coli*. However, only the 1992 review by Rajagopalan [81] put all these putative protein functions together and presented a pathway model where the single steps in Moco biosynthesis were indicated. They suggested for the first stage of Moco biosynthesis that GTP could be transformed into a sulfur-free pterin compound, the precursorZ (Figure 1), already possessing the Moco-typical four-carbon side chain, as shown in Figure 2a. Detailed labeling studies with *E. coli* [82] revealed evidence for a third unique route in pteridine synthesis, which starts from GTP with a novel and complex reaction sequence and results in the formation of precursorZ [83] as the first stable intermediate of Moco biosynthesis. PrecursorZ was suggested to be the substrate for the so-called “Converting Factor”, which transforms it to MPT. In contrast, the “Association factor FA” (a gene product of *chlB*) was situated at the end of the pathway subsequent to Moco formation (Figure 2a). Later, molecular analyses of cloned *chl* genes and the detailed biochemical analyses of the encoded proteins proved this working model to be correct for *E. coli*. Confusingly, the gene products of the *chl* genes became renamed in 1992 [28]; meanwhile, this new nomenclature has been accepted in the community. For more historical details about bacterial Moco biosynthesis, see the review by Leimkühler et al. [8].

### 2.7. 1992: A First Model for the Moco Biosynthesis Pathway in Plants

In 1992, I was appointed Professor of Botany at the Technical University of Braunschweig, and I formed a group to unravel Moco biosynthesis in plants on the molecular level. Initially, I summarized the comprehensive analyses of Moco mutants in plants involving genetic and biochemical studies outlined above, thus leading to the first working model of Moco biosynthesis [84]. The pterin-based structure of Moco and the presence of an alkyl side chain raised the possibility that its biosynthetic pathway could share steps or intermediates common to the biosynthesis of other pteridines. Several pteridines have three-carbon side chains, although Moco is unique in having a four-carbon atom side chain that forms the pyrano ring. For the plants, I had the following assumption (Figure 2b) [84]: because the pterin ring system also forms part of other essential compounds of metabolism (i.e., folic acid, flavines, and pteridines), the mutational block of its synthesis would be lethal for the cell. Two major pathways are known for synthesizing pterins and flavins involving the initial conversion of GTP by the enzymes cyclohydrolase I and II [85,86]. Therefore, I assumed that the products of the Cnx genes are more likely involved in the formation of the four-carbon side chain and the insertion of important sulfur atoms and the Mo atom (Figure 2b). For plants, I put the first common intermediate DHNPT (dihydro neopterin triphosphate) of the pterin pathway as a branchpoint where Moco biosynthesis could leave the common pterin pathway, and ordered the different Cnx proteins according to the biochemical characteristics found in their mutants. I positioned CnxA as putative Mo insertase as the last step. Later, the latter proved right and the former proved wrong (see above). Additionally, the Moco carrier protein’s existence should be considered because Moco was an unstable compound [84].

### 2.8. The 1990s: Moco Biosynthesis Was Uncovered in Pregenomic Times

In 1997, the *E. coli* genome was sequenced [87] followed by the human genome [88] and the *Arabidopsis thaliana* genome [89] in the year 2000. However, the molecular analysis of Moco biosynthesis in *E. coli*, plants, and humans started earlier than this, which I call the “pregenomic times”. Neither sequence databases nor internet-based search tools were available at that time. So how did we do it? Piece by piece, partial gene sequences were published for the *E. coli chl* genes in combination with fine structure mapping studies. As early as 1987, Reiss et al. [90] cloned a genomic fragment, indicating that there might be at least seven complementation regions (putative genes) that were able to complement mutants in *chlA*, *chlB*, *chlE*, and *chlG*. One year later, Nohno et al. [91] cloned and sequenced two open reading frames for the *chlE* locus. In 1993, Rivers et al. [92] presented the molecular analysis of the complete *chlA* locus, which was renamed as the *moaA* operon. These efforts formed the basis for characterizing the corresponding proteins encoded by these genes.

**Figure 3:**
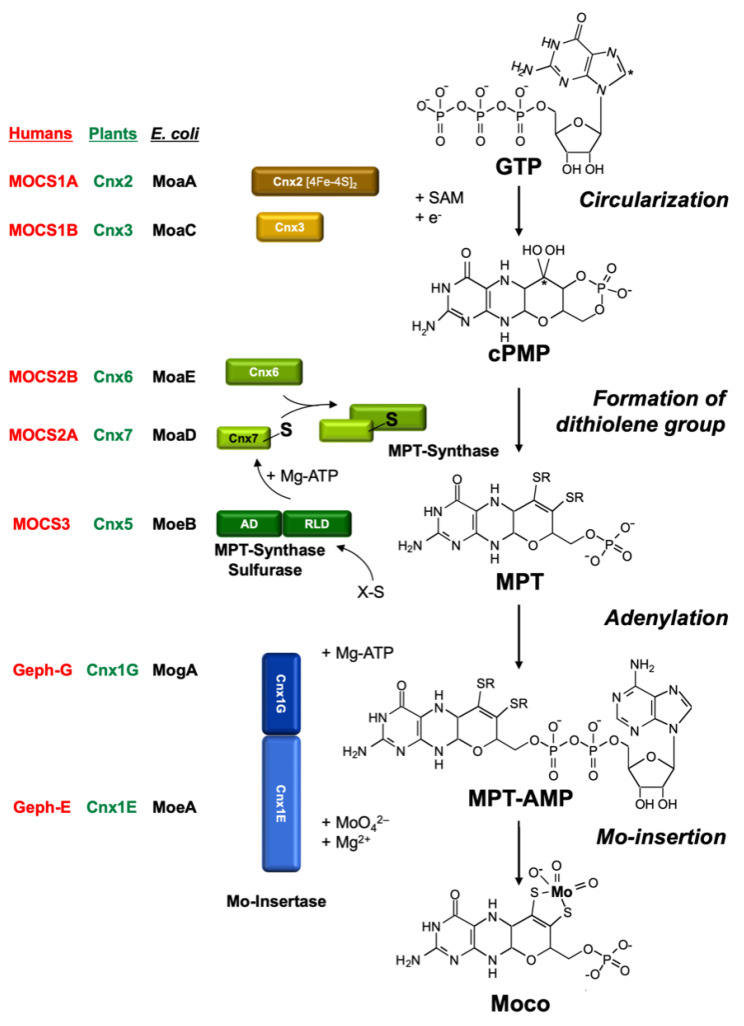
Pathway of Moco biosynthesis in eukaryotes. The pathway can be divided into four steps, each being characterized by its main features as given in italics on the right side. The names for the proteins from the plant *Arabidopsis thaliana* (green), humans (red), and *E. coli* (black) catalyzing the respective steps are given. In GTP, the C8 atom of the purine is labeled with a star. This carbon is inserted between the 2′ and 3′ ribose carbon atoms, thus forming the new C1′ position in the four-carbon side chain of the pterin (labeled with a star in cPMP). The in vivo sulfur (X-S) source for Cnx5 and MOCS3 is probably cysteine. Steps three and four in eukaryotes are catalyzed by the individual domains of Cnx1 (G and E) or Gephyrin (G and E). Functional properties such as [Fe–S] clusters in Cnx2 and Mocs1A, the use of S-adenosyl methionine (SAM), adenylation, and sulfuration of the small subunit of MPT synthase (Cnx7 and Mocs2B, respectively) are indicated. In Cnx5, MoeBD denotes the MoeB-like domain and RLD the rhodanese-like domain. Modified after Mendel and Kruse [93].

The MPT structure of Moco is conserved in all organisms. Hence, it was tempting to conclude that perhaps part of the biosynthetic pathway for Moco could also be similar in all organisms other than *E. coli*, perhaps even in higher organisms such as humans and plants. The first attempts to clone eukaryotic Moco genes using the *E. coli* genes as hybridization probes failed. Today, we know that the homologies on the DNA level are not sufficient for this approach. Hence, the following problem arose: How to clone a gene for which there is only a corresponding mutant? Furthermore, it had to be considered that the gene product of a given Moco gene is not Moco itself, but rather a protein involved at some step in Moco biosynthesis. We have successfully used three approaches to clone eukaryotic genes involved in Moco biosynthesis: (1) the functional complementation of *E. coli* Moco mutants; (2) the generation of Moco-defective plant mutants by tagging; and (3) exploiting protein–protein interactions using the yeast two-hybrid system. Most of these genes showed only negligible homologies to the *E. coli* genes on the DNA level. However, on the amino acid level, significant homologies (30–40% identity) did emerge between bacterial and eukaryotic proteins. It turned out that all *E. coli* Moco proteins (except *MoaB* and *MobAB*) have counterparts in plants, fungi, and humans, as shown in Figure 3. However, in addition to these homologies to the bacterial proteins, there are also differences typical for eukaryotes.

Most of our initial knowledge about eukaryotic Moco biosynthesis is derived from plant studies. This may be surprising; however, in pre-genomic times, the detailed characterization of NR-deficient mutants (using callus cultures and in vitro plants) substantially contributed to our understanding of the genetics and biochemistry of Moco biosynthesis (summarized by Müller and Mendel [78]). These NR-deficient mutants fell into two groups: those with a defect in the NR apoprotein, and others which were defective in Moco. The identification of six genetic complementation groups among Moco-deficient plant mutants in the 1980s (using crossing methods and protoplast fusions) and the ongoing molecular gene analysis in *E. coli* in the 1990s also laid the basis for cloning plant and human genes. In 1995, the first eukaryotic Moco biosynthesis genes were cloned and published for the plant *Arabidopsis thaliana,* followed by the first human gene in 1998. subsequently, I will reflect on the history of this molecular and biochemical endeavor in the order of the three steps of Moco biosynthesis. For comparison, Figure 3 gives the names of proteins involved in Moco biosynthesis in plants, humans, and *E. coli*, and the pathway of Moco biosynthesis is shown.

### 2.9. Molecular and Biochemical Analysis of Moco-Biosynthesis in Plants and Humans—The Early Years

#### 2.9.1. Step 1: Conversion of GTP to PrecursorZ (cPMP)

PLANTS: In 1995, the genes for the *Arabidopsis thaliana* proteins Cnx1, Cnx2, and Cnx3 (Figure 3) were cloned by functional complementation of the *E. coli* Moco mutants *mogA, moaA,* and *moaC* [94,95], respectively, which indicates that the functions of these proteins have been strongly conserved during evolution. Cnx2 and Cnx3 are involved in the first step of Moco biosynthesis, whereas Cnx1 catalyzes the last step. Nevertheless, a consortium for the nomenclature of plant genes [96] assigned Cnx1 as the number 1 as the function of this protein was best characterized in the preceding years. No biochemical data were available for Cnx2 and Cnx3 at that time.

More than one decade later, my postdoc Florian Bittner and his team discovered that step 1 of plant Moco biosynthesis occurs in the mitochondria, where GTP is available as a substrate for precursorZ synthesis and where Fe–S clusters are synthesized as the essential prosthetic group for Cnx2 [97]. Both proteins Cnx2 and Cnx3 contain a mitochondrial targeting sequence. After its synthesis in mitochondria, PrecursorZ is exported to the cytosol, where all further reactions occur. From now on, I will refer to PrecursorZ with the name “cyclic pyranopterin monophosphate (cPMP)”, which we assigned to this compound after having characterized it in greater detail [98,99] as a geminal diol. This geminal diol served as a matter of discussion that Günter and I later reviewed in detail [100].

Florian Bittner used a mutant of the transporter protein Atm3, which is localized in the inner membrane of mitochondria. However, when the Atm3 exporter is mutated, cPMP accumulates within mitochondria, and the cytosol becomes depleted; consequently, Moco levels and Mo enzyme activities decrease in the cell. The human analog to Atm3 is designated ABCB7.

HUMANS: As the gene sequences for the plant proteins Cnx2 and Cnx3 were known, they were taken as probes to identify the corresponding human genes, because we assumed that Moco biosynthesis is a highly conserved pathway, which turned out to be correct. Surprisingly, these two proteins are encoded by only one gene (*mocs1*) [101]. The corresponding transcript is bicistronic, with two consecutive reading frames separated by a stop codon. The first reading frame encodes for MOCS1A and the second one for MOCS1B. Initially, we tried to name the human genes “mcs”, for “Moco synthesis”, but this gene symbol was already assigned for a muscle gene, so we chose “mocs”. No biochemical data were available for the MOCS1 proteins at that time.

#### 2.9.2. Step 2: Conversion of PrecursorZ (cPMP) to MPT

PLANTS: In the 1990s, the *E. coli* MPT synthase was characterized to consist of a small and a large subunit where the small subunit carries a sulfur atom at its highly conserved C-terminus, which is transferred to cPMP. This series of papers is summarized by Leimkühler et al. [8]. However, there was no way to clone eukaryotic cDNAs encoding for MPT synthases using the approach of functional reconstitution in *E. coli*, as had worked successfully for the plant genes Cnx1 [94], Cnx2, and Cnx3 [95]. Therefore, BLAST N searches were performed in the Expressed Sequence Tag Database (GenBank) to pull the corresponding plant and human genes. Among eukaryotes (Figure 3), genes for MPT synthases were identified in plants, humans [102], and fungi [103]. Interestingly, they showed the same high degree of conservation in the C terminus of the small subunit with a typical double-glycine motif. In plants, the cloning of the small subunit of MPT synthase was achieved in a yeast two-hybrid screen using the assumed protein–protein interaction of the small subunit with the large subunit, exactly as we described this for the small subunit of the *E. coli* MPT synthase [104]. The result indicates that, in plants, both MPT synthase subunits interact to form MPT synthase. We named the large subunit of MPT synthase Cnx6 and the small subunit Cnx7.

HUMANS: Assuming that Moco biosynthesis is a highly conserved pathway, we used the amino acid sequence of the *E. coli* MoaE protein and performed a BLAST N search in the Expressed Sequence Tag Database (GenBank), and pulled the corresponding human gene that we named *mocs2* [102]. This human gene became even more exciting when we found out that it encodes for two proteins, namely the small and the large subunit of MPT synthase. On the bicistronic messenger RNA, the first reading frame codes for the small subunit MOCS2A and the second for the large subunit MOCS2B. This bicistronic gene disclosed another fascinating aspect because both reading frames overlap and exhibit a frameshift of +1 for *mocs2B*. Notably, we published this result in the honorable *American Journal of Human Genetics*, and I assume that we were the first plant biochemists to publish therein.

Bicistronicity ensures colinear expression and implicates the vicinity of the newly synthesized and interacting proteins. Such a micro-compartmentalization is certainly advantageous for low substrate concentrations, as in Moco biosynthesis. However, it remains enigmatic as to why in the human Moco biosynthetic pathway two times the extremely rare case of bicistronicity is found, whereas in higher plants, the corresponding genes are widely separated, and as in the case of step-1 proteins, are even located on different chromosomes. The plant cell needs the vicinity of newly synthesized and interacting proteins, because here, we encounter strong cytoplasmic streaming.

#### 2.9.3. Where Does the Sulfur Come from?

PLANTS and HUMANS: For *E. coli*, MoeB activates the small subunit of MPT synthase MoaD to form an acyl adenylate at the C–terminus in plants and humans, which is subsequently converted to a thiocarboxylate under the participation of the sulfurtransferase IscS [105,106]. This was the focus of Silke Leimkühler when she was a postdoc in the group of Rajagopalan. We used the MoeB gene sequence as a probe to pull the corresponding plant and human genes and named the encoded proteins Cnx5 and MOCS3, respectively [107]. To our surprise, the plant and human genes had N- and C- terminal extensions. No significant homologies were found for the N-terminal extension and no motifs emerged during database searches. However, the C-terminal extension forms a rhodanese-like domain encoded by a single exon. Rhodaneses are ubiquitous enzymes catalyzing the transfer of a sulfur atom. Thus, we assumed that this two-domain protein combines two functions: the adenylation of Cnx7/MOCS2A by its MoeB-like domain, and subsequent sulfur transfer by its rhodanese domain to form the thiocarboxylate at the C-terminus of Cnx7/MOCS2A. Experimental evidence for this was given by Silke Leimkühler when she joined my lab in 2001 as an independent group leader. She showed that the rhodanese domain of human MOCS3 transfers a sulfur atom to MOCS2A [108,109], and that this thiocarboxylation is of functional importance for humans [110]. Silke left my lab in 2004 to take up a professor position at the University of Potsdam. In summary, evolution combined the two functions “activation” and “sulfur transfer” catalyzed by two separate proteins in *E. coli* in one protein in eukaryotes.

### 2.10. Step 3: Formation of Active Moco—The Insertion of MO into MP

In plants, after synthesis of the MPT moiety, the chemical backbone is built for binding and coordinating the Mo atom. Mo is taken up into the cell in the form of molybdate, which is facilitated by high-affinity transport systems (reviewed by Minner-Meinen et al. [111] in this Special Issue). As described above in the paragraph “High molybdate repair”, molybdate-repairable mutants have been known for a long time: *Aspergillus* CnxE [63], *Neurospora nit–9ABC* [112,113], and tobacco CnxA [40]. The gene defective in the molybdate-repairable *Arabidopsis thaliana* mutants is Cnx1. My Ph.D. student, Birgit Stallmeyer, isolated it by functional complementation of the molybdate-repairable *E. coli mogA* mutants [94]. Surprisingly, the encoded protein Cnx1 was much larger than *E. coli* MogA. It consists of two domains (Figure 3) where the N-terminus (we named it E-domain) is homologous to *E. coli* MoeA and the C-terminus (we named it G-domain) is homologous to *E. coli* MogA. The high functional homology allows Cnx1 or its separately expressed G domain to replace the bacterial MogA function very efficiently [114]. In contrast, the N–terminus of Cnx1 was not able to take over the task of its homologous bacterial protein MoeA.

A protein that acts as Mo insertase should bind both substrates, Mo and MPT, to convert MPT into active Moco. Günter Schwarz, who was a Ph.D. student in my group at that time, purified the two domains of Cnx1 separately and showed a high-affinity interaction between MPT and Cnx1G (K_d_ = 100 nM); additionally, the Cnx1G domain was able to bind MPT (K_d_ = 1 µM) [115]. These data suggest a common binding fold for MPT in both domains of Cnx1. After graduation in 1998, Günter stayed in my group and started his own team. To identify functionally important residues within Cnx1G, he performed a mutagenesis screening followed by selection for loss of function using *E. coli mogA* complementation [116], thus prompting the assumption that MPT binding is not the only function of Cnx1G. Rather, it should activate MPT in an as-yet unknown way [116]. Four years later, with the discovery of MPT–AMP bound and generated by Cnx1G [117], this assumption proved to be correct. Günter also assumed that the E-domain of Cnx1 is essential for activating Mo and inserting it into MPT, thus forming active Moco [116]. Here, our key question was whether molybdate serves as a donor for insertion of Mo into MPT or whether molybdate has to undergo intracellular processing prior to insertion. Günter left my group in 2004 and took up a professor position at the University of Cologne. However, in 2006, we believed that molybdate had to be activated (by ATP) to be ready for insertion in MPT [118]. For a decade, we did not touch Cnx1; then, my former Ph.D. student, Tobias Kruse, who meanwhile had set up his own team in my group, restarted Cnx1 research. His team determined the atomic structure of the E-domain [119] and revised our old model by showing that no activation of molybdate was necessary for insertion into MPT [120,121]. Details are given in Tobias Kruse’s review in this Special Issue.

In 1999, Birgit Stallmeyer was about to graduate in my group. Previously she had cloned and characterized the gene for Cnx1 in humans [94]. While Günter Schwarz continued to elucidate the biochemistry of Cnx1, Birgit became interested in studying human Moco biosynthesis, and she not only identified the bicistronic *mocs2* gene [102], but also found the mammalian counterpart to the plant Cnx1 Mo-insertase, the protein Gephyrin. Gephyrin was previously described as a neuroreceptor anchor protein linking glycine receptors in the postsynaptic membrane to the subcellular cytoskeleton [122]. Gephyrin is thought to be an instructive molecule for the formation of glycinergic synapses, and its expression was shown to be essential for the postsynaptic aggregation of glycine receptors [123]. Based on the homology of Gephyrin to Cnx1, we suggested an additional function of Gephyrin in Moco biosynthesis. Birgit showed that recombinant Gephyrin binds MPT with high affinity, heterologous Gephyrin expression could restore Moco biosynthesis in *E. coli*, plants, and the murine cell line L929, and the inhibition of Gephyrin expression by antisense RNA expression in cultured murine cells significantly reduced their Moco content [124]. In addition, Gephyrin knockout mice did not only show the expected absence of synaptic glycine receptor clustering, but also developed symptoms identical to those of Moco deficiency [125] where no SO activity could be detected. It is obvious that Gephyrin combines two different functions, such as (1) biosynthetic activity in Moco formation and (2) a structural role in receptor clustering. The latter function is evolutionarily younger, and hence must have been recruited from the older in primary metabolism. Notably, in Gephyrin, the orientation of the two domains is reversed—it has the E-domain on its N-terminus and not the G-domain as in Cnx1. The same orientation was seen in the Gephyrin-homologous protein Cinnamon in the fly *Drosophila melanogaster* [126], and this orientation was also described in the corresponding fungal protein *Neurospora crassa* Nit-9 [127]. There were two independent evolutionary events, one for the animal lineage in the Gephyrin orientation and one for the plant lineage in the Cnx1 orientation. Interestingly, the Gephyrin linker between the E- and G-domains is much longer than in Cnx1 (134 vs. 11 amino acids). After starting his group at the University of Cologne, Günter Schwarz focused on Gephyrin research. We now know that the long linker in Gephyrin represents a separate domain involved in the modulation of signaling pathways [128]. On another note, there must have been evolutionary pressure to fuse monofunctional bacterial proteins to multifunctional eukaryotic proteins, perhaps to facilitate a better substrate-product channeling [128].

### 2.11. Cytoskeleton Binding

**Figure 4:**
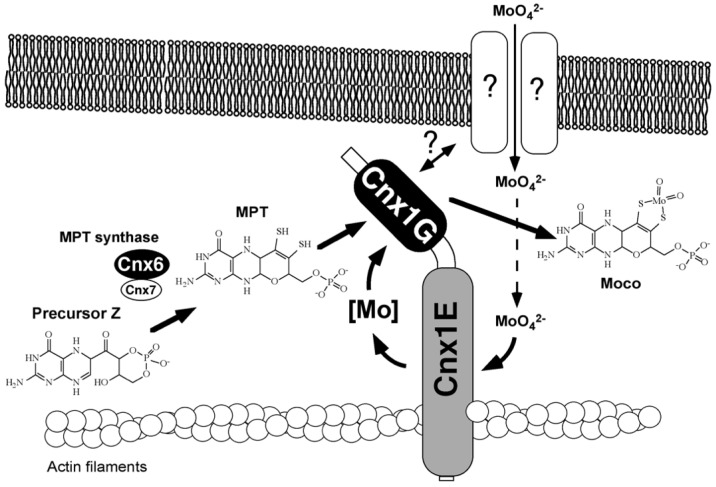
Model for the Moco biosynthesis pathway in plants, published by Schwarz et al. in the year 2000 [129]. Cnx1 is shown to be bound to an actin filament. An unidentified molybdate transport system was proposed that interacts with Cnx1 to facilitate molybdate channeling to the E domain, given that a mutation in this protein results in a molybdate-repairable phenotype. The conversion of precursorZ cPMP) to MPT by the MPT synthase (Cnx6 and Cnx7) is shown. MPT is highly sensitive to oxidation; therefore, it was suggested that the rapid conversion of precursorZ to Moco should occur in a multienzyme complex anchored by Cnx1 on the cytoskeleton. Modified after Schwarz et al. [129].

Gephyrin fulfills its anchoring function for neuroreceptors by linking them via its E-domain to actin filaments of the subcellular cytoskeleton [123]. Gephyrin and its plant homolog Cnx1 exhibit functional properties that are distinct from Moco biosynthesis. Based on the observed cytoskeleton binding of Gephyrin, in vitro experiments demonstrated binding to actin filaments for Cnx1 [129], and I wanted to demonstrate this in the living cell. Robert Hänsch, who conducted his Ph.D. in our group, stayed with me and founded his own team. He set up live-cell imaging using confocal laser scanning microscopy, and later, he confirmed the cytoskeleton binding of Cnx1 in the living cell [130]. In 2000, Günter Schwarz’s finding of cytoskeleton anchoring prompted speculations on whether this might be of functional importance [129]. One reason we saw was the anchoring and stabilization of a putative multienzyme protein complex for Moco-biosynthesis. The other reason was the spatial positioning of Cnx1 next to a putative molybdate importer for sequestering incoming molybdate into molybdate insertase Cnx1. Figure 4 presents our early model published in 2000 [129]. Years later, both assumptions were proven by live-cell imaging in Robert Hänsch’s team [130,131]. In this old model, we assumed that the G-domain was inserting molybdate because mutants in the G-domain could be repaired in vivo by high levels of molybdate. We had not yet discovered MPT–AMP on the G-domain (which was only in 2004)—nowadays, we know that it is the E-domain that catalyzes the insertion of molybdate into MPT supplied by the G-domain in the form of MPT-AMP. Robert Hänsch’s team found that Cnx5, Cnx6/7, and Cnx1 (catalyzing steps 2 and 3) undergo tight protein–protein interactions in the cytosol of living plant cells [132], thus supporting the idea of channeling the fragile Moco intermediates in a protected way within a transient multiprotein complex. Furthermore, Cnx1 also makes protein–protein contact with the molybdate transporter in the living cell. Details are summarized in the paper by Minner-Meinen et al. [111] in this Special Issue.

### 2.12. Bacteria Have Additional forms of Moco

As first detected by Krüger and Meyer in 1986 [133,134], bacteria can carry various substituents at the C4′ atom of the side chain of Moco. Johnson et al. [135] showed that the Moco from DMSO reductase from *Rhodobacter sphaeroides* consists of an MPT guanine dinucleotide where a GMP is bound to the C4′ atom via a pyrophosphate bond. Other prokaryotic variants of the cofactor containing CMP, AMP, or IMP linked to the MPT were also identified [81]. These dinucleotide forms were only found in prokaryotes. Bacteria may even contain both Moco forms, because it was found that XDH from *Rhodobacter capsulatus* and *Pseudomonas putida* contains the MPT form of the cofactor, whereas the other Mo enzymes (NR, DMSO reductase, and chinoline oxidoreductase) contain the dinucleotide form [8,136]. Another difference between bacterial and eukaryotic Moco biosynthesis is the formation of bis-MPT-based cofactors in bacteria where two dithiolenes of two MPT molecules coordinate one Mo (or W) atom. Details of the role of these nucleotide attachments to MPT are discussed by Tiedemann et al. [52] in this Special Issue. For a comprehensive review of Moco biosynthesis and insertion into Mo enzymes, see Magalon and Mendel [137].

### 2.13. Terminal Sulfuration of Moco

**Figure 5:**
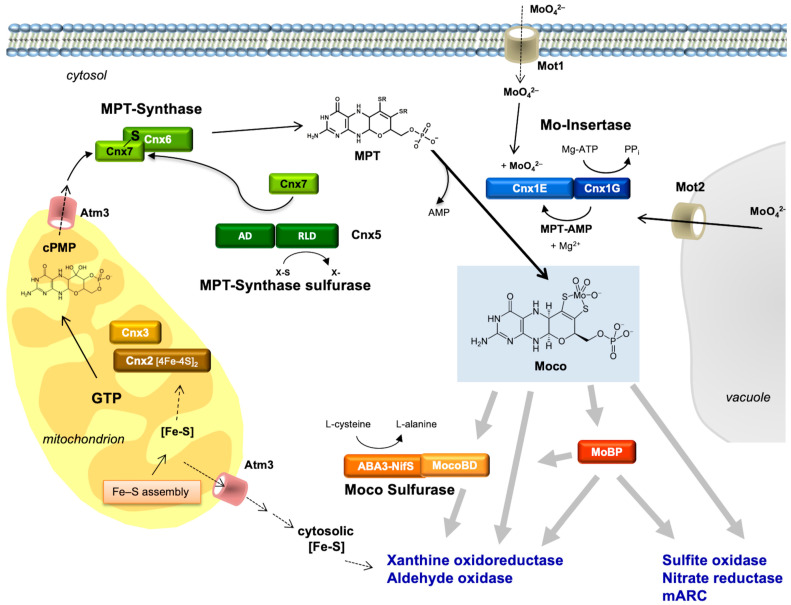
Present model for the biosynthesis, distribution, and maturation of Moco in plant cells. Biosynthesis starts with the conversion of GTP to cPMP in the mitochondria. The dependence of Cnx2 on [Fe–S] is indicated. The transporter Atm3 is assumed to be involved in the export of cPMP to the cytosol [97], where MPT-synthase, consisting of Cnx6 and Cnx7, is sulfurated by Cnx5, with the primary sulfur donor of Cnx5 (X-S) being unknown. The individual reactions of the Mo insertase Cnx1 and its products (Moco, pyrophosphate, and AMP) are indicated. Molybdate for this reaction is supplied by the vacuolar exporter Mot2 (a Mot2-mutant leads to a shortage of molybdate in the cytosol and the accumulation of molybdate in the vacuole) [146]. Later, Mot2 was renamed as Mot1.2 [111]. The Mot1-transporter is the cellular Mo-importer and resides in the plasma membrane [111]. Mature Moco can be either bound to a Moco-binding protein (MoBP) or directly to the Mo enzymes. The Moco sulfurase ABA3 generates a protein-bound persulfide, which is the source of the terminal sulfur ligand of Moco enzymes in the XDH/AO family. Similarly to Cnx2, xanthine dehydrogenase and aldehyde oxidase also depend on [Fe–S] from mitochondria. Modified after Mendel and Kruse [93].

As a final step of maturation, the eukaryotic xanthine dehydrogenase and aldehyde oxidase as enzymes with a mono-oxo Mo center require the addition of a terminal inorganic sulfide to the Mo site. This sulfur ligand does not originate from the apoprotein, nor does it come from the Moco moiety. For rats and flies, the early work of Wahl and Rajagopalan [42] demonstrated that, in vitro, this sulfur can be spontaneously lost or can be removed from the enzymes by cyanide treatment, generating an inactive enzyme. The reaction, however, is reversible, and the enzyme can be reactivated by sulfide treatment under reducing conditions. In vivo, this terminal sulfur has to be added by a separate enzymatic reaction catalyzed by a Moco sulfurase (Figure 5). Moco sulfurase activities were earlier described for *Drosophila melanogaster* Ma–l [138], the fungus *A. nidulans* HxB [139], cattle [140], humans [141], and plants (ABA3 [142]). The N-terminus of these proteins shares significant homologies with the bacterial sulfur transferase NifS. Mutants defective in this Moco sulfurase were lacking both xanthine dehydrogenase and aldehyde oxidase activities, but retained their activities of enzymes with a dioxo Mo center, such as NR in plants [139,141] and SO in cattle and insects [138]. In both the fly *ma–l* and the fungal *hxB* genes, intragenic complementation was observed [143], demonstrating that the N-terminal NifS-like domain and the C-terminal domain function independently within the oligomeric sulfurase protein. In 2001, my Ph.D. student Florian Bittner cloned and characterized the plant Moco sulfurase ABA3 from *Arabidopsis thaliana* [144]. He characterized the N-terminal domain as cysteine desulfurase. He showed in a defined in vitro system that purified ABA3 was able to activate aldehyde oxidase, using L-cysteine as a sulfur donor. Thus, we suggested that the C-terminal domain recognizes the target enzyme and catalyzes a trans-sulfuration reaction to introduce sulfur into Moco. As a postdoc, Florian set up his own team in my group and determined the mechanism of ABA3-catalyzed Moco sulfuration (as summarized in [145]).

### 2.14. Moco Carrier Proteins

Free Moco is sensitive to oxidation; therefore, it was assumed that Moco permanently occurs as protein-bound in the cell. We assumed that either apo-Mo enzymes connect directly to Cnx1-E to receive Moco, or Moco has to become bound to a carrier protein that protects and stores Moco until further use, thus providing a way to buffer the supply and demand of Moco. Therefore, a cellular Moco distribution system should meet two demands: (i) it should bind Moco subsequent to its synthesis, and (ii) it should allocate Moco from the Moco donor Cnx1E to the Moco-dependent enzymes. In 1992, a Moco carrier protein (CrMCP) was identified in the green alga *Chlamydomonas reinhardtii*, which was found to tightly bind Moco, thus protecting it against oxidation [147]. We showed that without any denaturing procedure, the subsequent transfer of Moco from CrMCP to apo-nitrate reductase from *Neurospora crassa* was possible. These properties of Chlamydomonas MCP made it a promising candidate for being part of a cellular Moco delivery system. In 2006, Günter Schwarz’s team solved the crystal structure of CrMCP. It is a homotetramer with each 16 kDa monomer binding one molecule of Moco and possessing a typical Rossmann fold [148]. However, this crystal structure was empty, i.e., it had no co-structure with Moco. One decade later, in 2016, Jörn Krausze, a crystallographer who joined my group’s team of Tobias Kruse, tried hard to co-crystallize CrMCP with Moco; however, was also unsuccessful. We could solve the co-structure of the MCP with Moco after switching from Chlamydomonas to another green algae (*Volvox carterii*). Details are given in the review by Tobias Kruse in this Special Issue. Tobias Kruse not only investigated Mo insertion into MPT (see chapter above), but in 2010, during his Ph.D. time in my group, discovered a novel protein family in the plant *Arabidopsis thaliana* consisting of eight members that can all bind Moco, which are therefore named Moco-binding proteins (MoBPs) [149]. These proteins bind Moco less tightly than Chlamydomonas MCP and are therefore no good candidates to serve as Moco storage proteins. Rather, they seem to be involved in the cellular distribution of Moco.

In summary, these carrier proteins only occur in some bacteria, algae, and in higher plants, but not in fungi and mammals. None of them was identified via a selectable loss-of-function mutant phenotype. Hence, their function is not mandatory for Mo enzyme maturation. Instead, they may contribute to cellular Moco allocation.

### 2.15. Moco Deficiency in Humans Is Lethal

Moco deficiency is a rare recessive inborn error of metabolism first described by Duran in 1978 [150], resulting in a complete loss of activities of all Mo enzymes, which is lethal for the patient. After cloning all human Moco biosynthesis genes, Günter Schwarz and I decided to develop a Moco replacement therapy for this devastating disease. Moco itself and MPT were exceedingly unstable, but cPMP was sufficiently stable [98]. Therefore, we joined forces with the geneticist Jochen Reiss from Göttingen University, who had performed molecular patient analysis and had shown that type A patients exhibit a mutation in *mocs1* and are unable to synthesize cPMP. These patients are most prevalent. Type B patients accumulate cPMP due to a mutation either in *mocs2* or *mocs3* (resulting in the loss of MPT synthase activity). Type C patients are extremely rare and carry a mutation in *gephyrin* [151]. These genetic analyses confirmed prior biochemical characterizations of mutant cell lines by Vivian Shih. In 1989, she made a breakthrough observation that became the compass for our own research strategy. Vivian Shih used cell cultures of numerous Moco-deficient patients and biochemically defined two groups of patients. Group A cells were unable to synthesize cPMP; group B cells could not convert cPMP into MPT. Most excitingly, she co-cultured cells from groups A and B and observed the reappearance of sulfite oxidase activity. She concluded that “a relatively stable diffusible molybdopterin precursor produced by group B cells and utilized by group A cells to synthesize active molybdenum cofactor is identified, and evidence is presented which suggests that this diffusible intermediate is identical to a molybdopterin precursor…” [152]. Thus, cPMP was excreted from one type of cells and taken up by the other type. This was the first and most important indication that replacement therapy with cPMP should be feasible. However, in my laboratory, Günter Schwarz’s team developed a mouse model for step 1 Moco deficiency resembling the patient phenotype. We successfully cured these mice with an intrahepatic injection of cPMP, restoring Mo enzyme activity and lifespan extension [153]. cPMP was purified from an overproducing *E. coli* strain that they had developed. After Günter left my group, he focused on the clinical translation of this therapy. His first patient therapy was reported in 2010 [154]. For details of this endeavor, see his review in this Special Issue. In 2021, the FDA approved cPMP therapy, and chemically synthesized cPMP is marketed under the name Nulibry.

### 2.16. Is There Life without Moco?

All higher organisms possess Mo enzymes. However, genome-wide database analyses revealed a significant number of bacteria and unicellular lower eukaryotes (yeasts, fungi, and protists) that do not need Mo. This suggests that Mo utilization is an ancient trait and was once common to almost all species in the three domains of life. As a matter of fact, Mo utilization was already present in LUCA, the last universal common ancestor of life [3]. Large-scale comparative genome analyses revealed that among eukaryotes, about 30% of all species had lost Mo utilization. They were mainly unicellular yeasts, fungi, and protists [155]. It turned out that there was a high correlation between the loss of Mo utilization and habitat and lifestyle [156]. The majority of obligate intracellular parasites and symbionts lost the ability to use Mo and exhibited condensed (i.e., shortened) genomes, which are associated with their intracellular parasitic life and the evolutionary pressure on genome size—nevertheless, these organisms may still use Mo-dependent enzyme functions of their host. All multi-cellular eukaryotes are dependent on Mo [157]. Model yeasts such as *Saccharomyces cerevisiae* and *Schizosaccharomyces pombe* played no role in Mo research, because they belong to those organisms that do not contain Mo enzymes. In contrast, other yeasts such as *Pichia pastoris* do need Moco. However, there is an indication that *Saccharomyces cerevisiae* should have possessed Moco biosynthesis, because a MoeB-homologous protein sequence occurs in the highly conserved ubiquitin-activating enzyme E1 (Uba4) in eukaryotes using the same adenylation mechanism as in MoeB. This finding suggests that protein E1 derived from the ancestral gene moeB [158] which, in *Saccharomyces cerevisiae*, was lost along with all other members of Mo metabolism during evolution.

## 3. Conclusions

Moco is an ancient invention of nature. The structure of MPT as the organic moiety of Moco is highly conserved. In bacteria, a nucleotide is attached to MPT and MPT can form dimers. Moco occurs ubiquitously—except for a number of bacteria, fungi, and unicellular yeasts that have lost Mo metabolism. During the evolution from prokaryotes to eukaryotes, the pterin core structure of Moco has not changed. Accordingly, its biosynthetic pathway is conserved, and diverse genome projects have revealed a similar set of Moco genes in bacteria, archaea, fungi, flies, plants, and mammals. However, there are also some notable differences between prokaryotes and eukaryotes. During evolution, the genes of single monofunctional prokaryotic proteins have been fused in some cases to form two-domain multifunctional eukaryotic proteins, as in Arabidopsis Cnx1 and animal Gephyrin (comprising *E. coli* MogA and MoeA). Furthermore, the dinucleotide form of Moco has not been found in eukaryotes. Another difference resides in the complex morphology and size of a eukaryotic cell with its several compartments, thus making adaptations of the Moco biosynthetic pathway inevitable, as in the case of the cytoskeleton binding.

For all Moco-using organisms, Moco is essential, and a defective Moco has detrimental or lethal consequences due to the pleiotropic loss of all Mo–enzymes. To this end, the single steps of Moco biosynthesis were uncovered in great detail, but the regulation of this pathway, as well as the steps how Moco is allocated and inserted into the apoproteins, are fascinating topics of ongoing research in several laboratories working with mammals, plants, fungi, nematodes, algae, and bacteria.

In 1974, I started studying Moco, and in 2021, I retired from my administrative and teaching duties, and I continue conducting Moco research. In collaboration with the young professor Kurt Warnhoff (USA), we study the unique case of the nematode *C. elegans*, which is able to take up protein-bound Moco from its diet and distribute it through its body until Moco reaches sulfite oxidase, where it becomes inserted into this essential enzyme [159,160]. Kurt discovered this phenomenon, and I contribute our biochemical knowledge to his project. Novel but still hidden mechanisms of Moco biology remain to be uncovered in this exciting emeritus endeavor into uncharted land.
